# Exploring the Excluded Stomach: A Case Series of Novel Endoscopic Techniques to Diagnose Gastric Cancer in the Excluded Stomach After Roux-en-Y Gastric Bypass Surgery

**DOI:** 10.7759/cureus.2825

**Published:** 2018-06-18

**Authors:** Saeed Ali, Abdelkader Chaar, Wesam Frandah, Rola Altoos, Zeeshan Sattar, Muhammad Hasan

**Affiliations:** 1 Internal Medicine Residency, Florida Hospital, Orlando, USA; 2 Internal Medicine, St. John Hospital and Medical Center, Detroit, USA; 3 Gastroenterology, University of Kentucky, Lexington, USA; 4 Diagnostic Radiology, Florida Hospital, Orlando, USA; 5 Internal Medicine, Khyber Teaching Hospital, Peshawar, PAK; 6 Gastroenterology, Florida Hospital, Orlando, USA

**Keywords:** endoscopy, bariatric, gastric, cancer, roux-en-y gastric bypass

## Abstract

Gastric cancer is the fifth most common malignancy worldwide and the fourth leading cause of cancer-related deaths. The diagnosis is usually made by direct visualization with supporting histopathology. However, patients with gastric bypass surgery pose a challenge in diagnosis due to the difficulty in the evaluation of the excluded stomach. We present two cases of gastric cancer in the excluded stomach after Roux-en-Y gastric bypass (RYGB) surgery was diagnosed using two different endoscopic approaches.

## Introduction

Roux-en-Y gastric bypass (RYGB) is one of the most commonly performed weight reduction surgeries. It has been performed quite frequently for morbid obesity since 1966 [1-2]. Evaluation of the diseases of the excluded stomach, such as ulcers or malignancy, poses a significant challenge due to the difficulty in accessing the excluded portion of the stomach (Ali S, Chaar A, Frandah W, Hasan MK: Exploring the excluded territory: a case of signet cell cancer of the excluded stomach - Poster P1686. World Congress of Gastroenterology at ACG2017 Mtg. Orlando, FL, Oct. 13-19, 2017. http://eventscribe.com/2017/wcogacg2017/ajaxcalls/PosterInfo.asp?efp=S1lVTUxLQVozODMy&PosterID=115969&rnd=0.6203457). Symptoms of gastric cancer in the excluded stomach are non-specific, such as weight loss, loss of appetite, and bloating. Physical examination findings may include epigastric tenderness or a palpable mass. Diagnosis is confirmed after imaging studies, such as computed tomography (CT) scan or endoscopic ultrasound (EUS). Upper gastrointestinal (GI) endoscopy is employed to get tissue diagnosis and localization of the primary tumor. Advanced endoscopic techniques, like antegrade double-balloon enteroscopy (ADBE), can be employed in difficult cases. A percutaneous route can be used with a combined radiologic and endoscopic technique in cases where conventional endoscopy is not successful. Surgery is the last resort after the failure of the conventional and combined endoscopic and percutaneous techniques. We report two cases where the diagnosis of gastric cancer of the excluded stomach was obtained using advanced endoscopy techniques.

## Case presentation

Case 1

**Figure 1:**
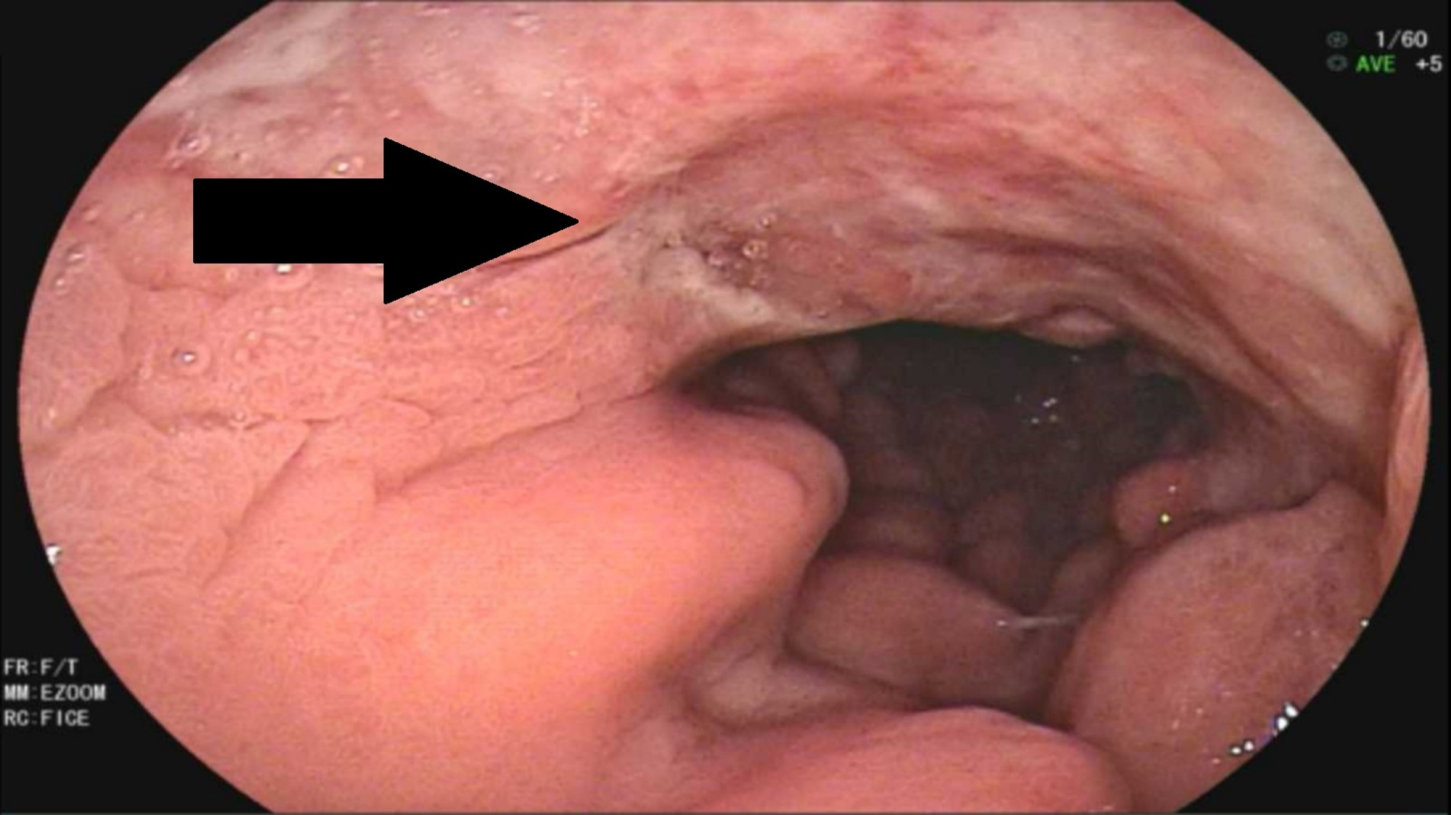
Endoscopic view of infiltrative ulcerated mass in prepyloric region and antrum of stomach (solid arrow).

A 40-year-old female with RYGB surgery performed 13 years ago for morbid obesity presented with epigastric pain and weight loss. She was recently diagnosed with bilateral metastatic signet cell carcinoma to the ovaries for which she was on chemotherapy. Her family history is significant for gastric cancer in her maternal aunt. CT imaging of the abdomen was unable to reveal the primary source of the tumor. Upper and lower GI endoscopies were unrevealing for malignancy with the upper endoscopy failing to examine the excluded stomach due to the limited length of the scope. The excluded stomach was subsequently approached using ADBE via the afferent limb of the Roux-en-Y bypass. A large infiltrative ulcerated circumferential mass was found in the prepyloric region and antrum of the stomach (Figure 1).

**Figure 2:**
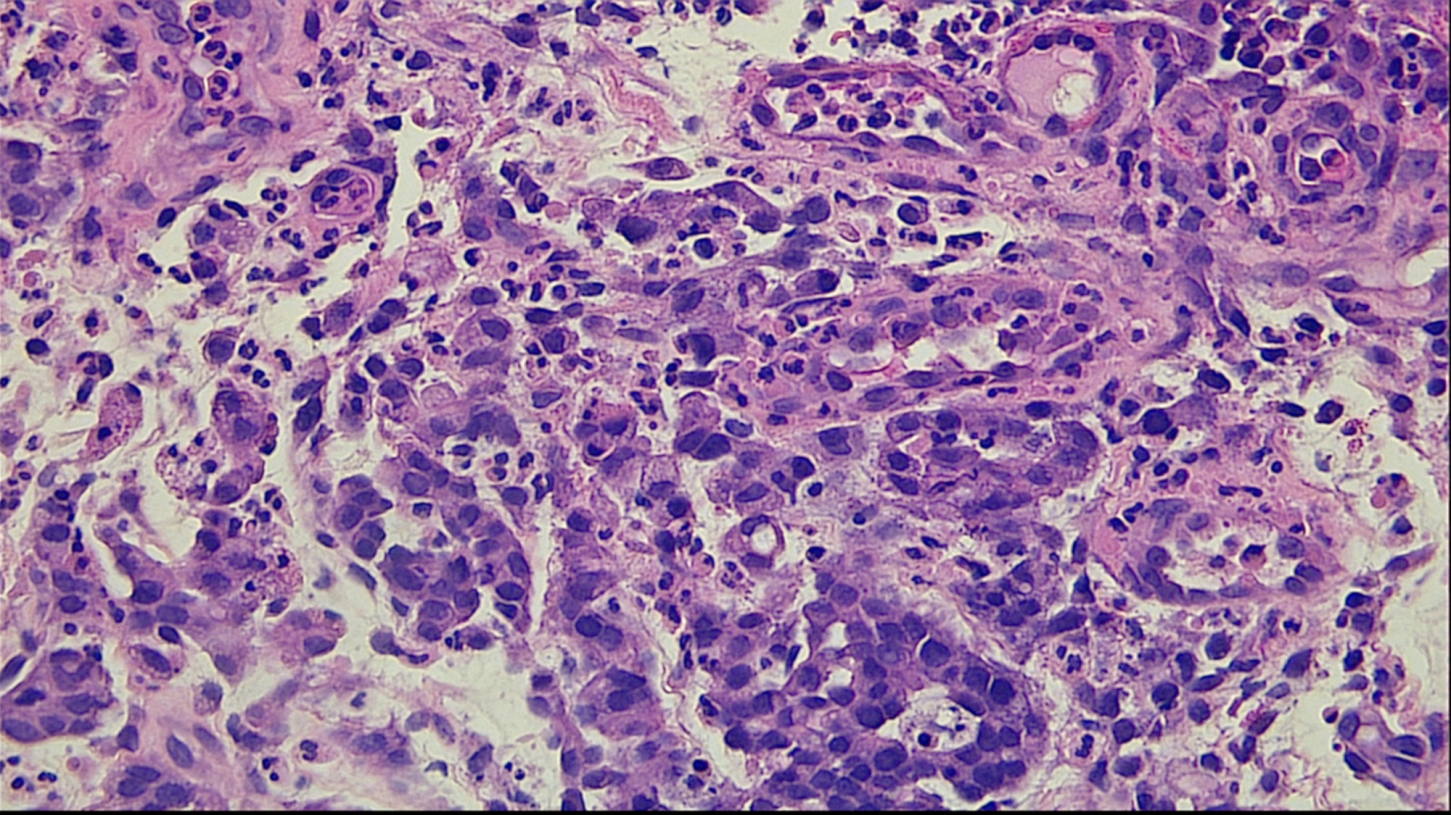
Histopathology slide showing signet cell gastric adenocarcinoma

Biopsies were negative for H. pylori infection and revealed invasive signet cell gastric adenocarcinoma (Figure 2) that likely had metastasized to the ovaries.

Molecular analysis showed a human epidermal growth factor receptor 2 (HER2)-negative tumor. The patient is currently undergoing chemotherapy for Stage IV gastric cancer with fluoropyrimidine, plus oxaliplatin (FOLFOX).

Case 2

**Figure 3:**
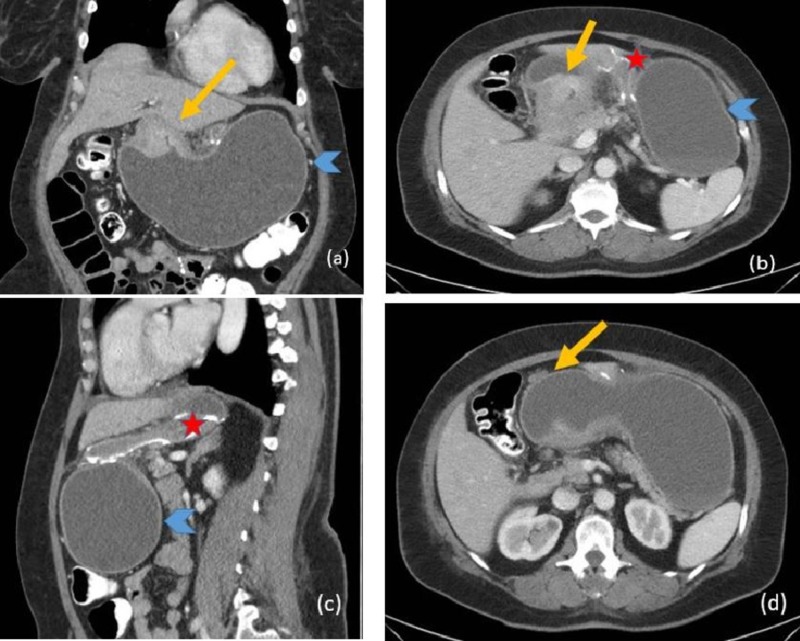
Abdominal computed tomography (CT) scan A-C show an obstructing, enhancing soft tissue mass at the pylorus measuring approximately 5.5 x 4.5 cm (yellow arrow). There is associated marked fluid-filled distention of the gastric remnant (blue arrowhead). Normal appearance of the Roux-en-Y gastric bypass (star). (D) Contrast-enhanced axial CT image at the level of the upper abdomen demonstrates a small soft tissue nodule anterior to the gastric antrum; indeterminate but suspicious for peritoneal metastatic disease.

A 50-year-old female with RYGB surgery performed six years previously presented with epigastric pain, nausea, and recurrent gastrointestinal bleeding of nine months duration. Multiple upper and lower GI endoscopies, as well as a video capsule endoscopy, failed to identify the source of her recurrent bleeding. A CT scan of the abdomen showed an obstructing enhancing soft tissue mass at the pylorus measuring approximately 5.5 x 4.5 cm (Figure 3, arrow) with marked fluid-filled distention of the gastric remnant and normal appearance of the RYGB. It also revealed a small soft tissue nodule anterior to the gastric antrum suspicious for peritoneal metastatic disease. 

**Figure 4:**
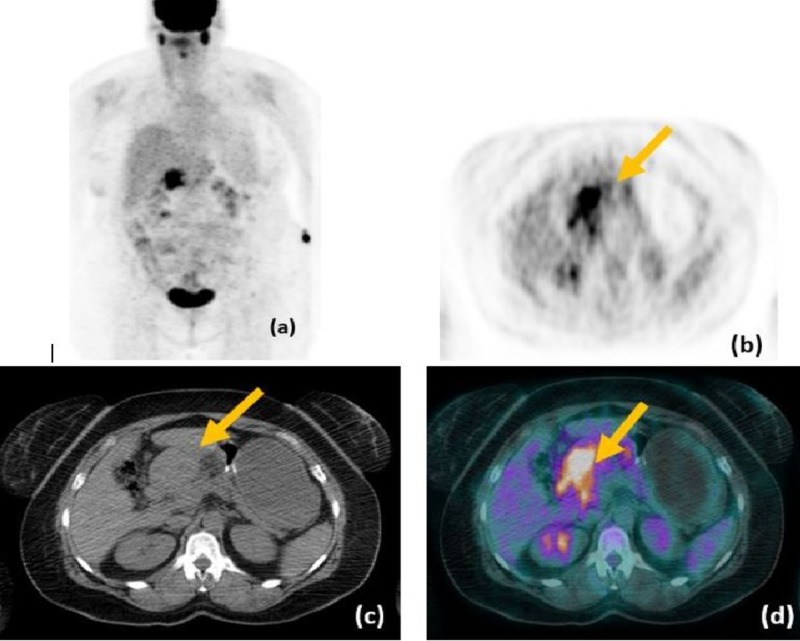
PET/CT scans A-D: Images from the same patient demonstrate marked hypermetabolism within the gastric pylorus mass. PET: positron emission tomography; CT: computed tomography

Positron emission tomography (PET)/CT images demonstrated marked hypermetabolism within the gastric pylorus mass; however, no evidence of fluorodeoxyglucose (FDG)-avid metastatic disease was identified (Figure 4).

**Figure 5:**
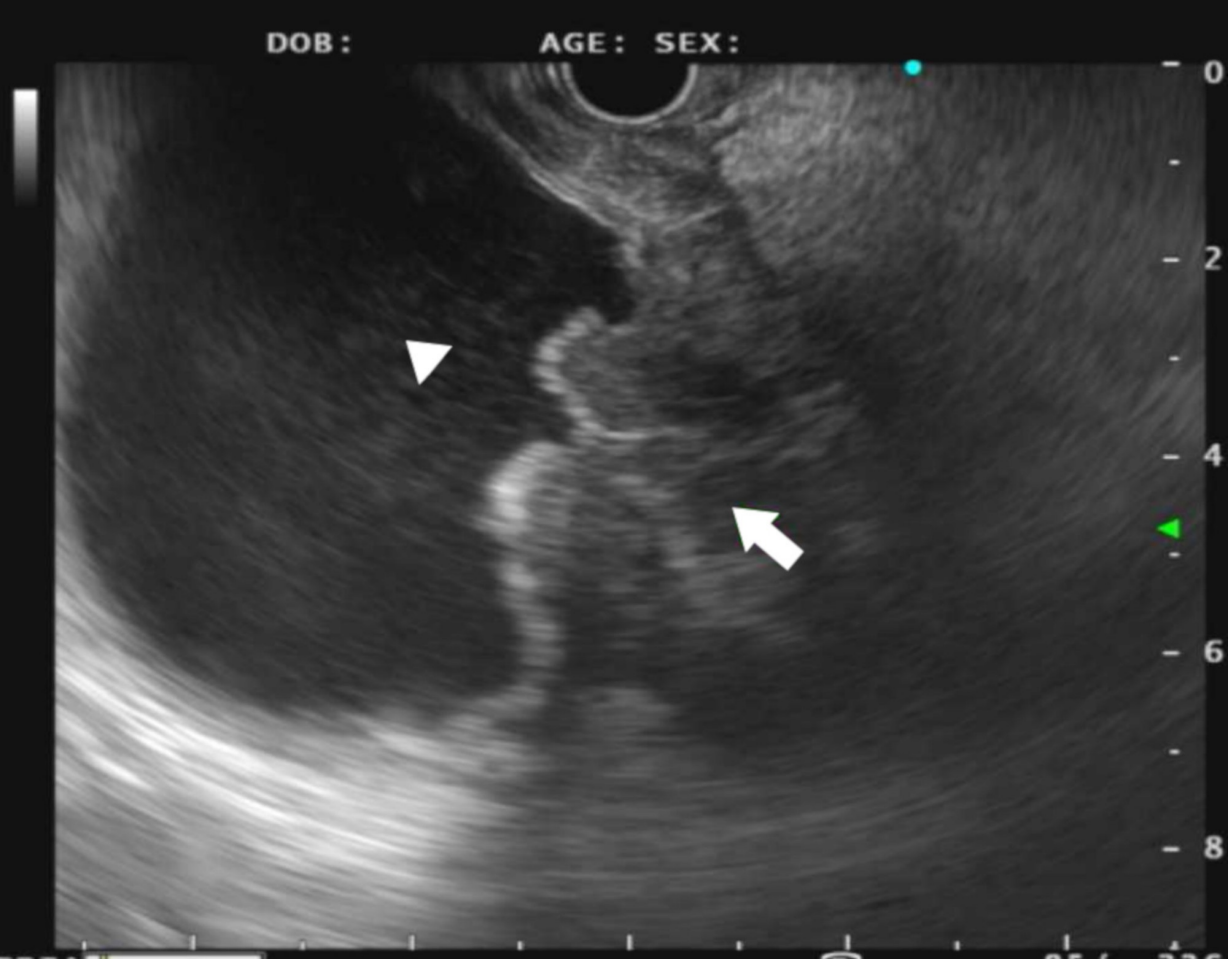
Endoscopic ultrasound (EUS) showing antral wall thickening in the excluded stomach (solid arrow)

Subsequently, an endoscopic ultrasound (EUS) via the gastric pouch showed diffuse wall thickening of the excluded stomach at the antrum, as well as two enlarged, hypoechoic, and well-defined lymph nodes in the gastrohepatic ligament (Figure 5).

**Figure 6:**
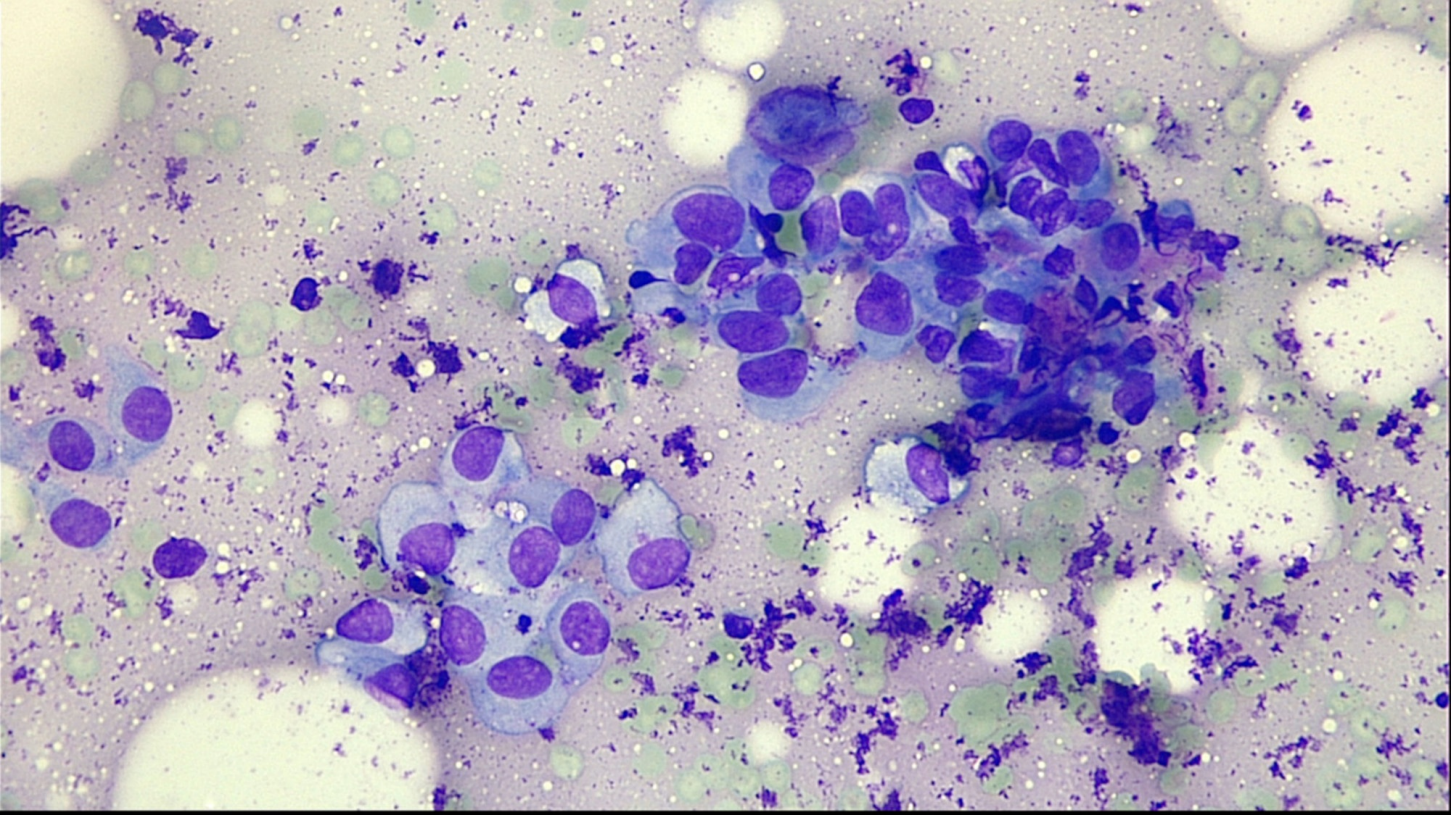
Histology slide showing poorly differentiated adenocarcinoma

Fine needle aspiration (FNA) of the lymph nodes and the gastric wall of the excluded stomach revealed poorly differentiated gastric adenocarcinoma (Figure 6).

The biopsy was negative for H. pylori infection. Molecular analysis showed a HER2-negative tumor. Staging laparoscopy confirmed peritoneal implants. The patient is currently undergoing chemotherapy for Stage IV gastric cancer with capecitabine, plus oxaliplatin (CAPOX).

## Discussion

Gastric cancer is the fifth most common malignancy worldwide and the fourth leading cause of cancer-related deaths. In 2012, about 952,000 new cases were diagnosed worldwide and 21,000 of them occurred in the United States compared to 162,000 in Europe [3]. RYGB surgery is one of the most effective weight reduction surgeries and involves the creation of a small proximal gastric pouch with a proximal closure of the remaining stomach and formation of a gastrojejunostomy with Roux-en-Y reconstruction [4]. Cancer in the excluded stomach is rare and only a few case reports have been reported [5]. One study reported 17 case reports describing 18 patients who underwent bariatric procedures. Adenocarcinoma of the excluded stomach was reported in six out of the seven patients who underwent RYGB surgery with the mean time to the diagnosis being 8.6 years (standard deviation (SD): 6.4 years) [6]. However, larger prospective studies are lacking regarding the incidence of cancer in this population.

Risk factors for gastric cancer include Helicobacter pylori infection, cigarette smoking, high-salt intake, family history of stomach cancer and a diet low in fruits and vegetables [7]. Similarly, obesity has been linked to an increased risk of developing cancer [8] (Ali S, Chaar A, Frandah W, Hasan MK: A novel endoscopic technique to diagnose gastric cancer in the excluded stomach after Roux-en-Y gastric bypass - Poster P1389. World Congress of Gastroenterology at ACG2017 Mtg. Orlando, FL, Oct. 13-19, 2017. https://eventscribe.com/2017/wcogacg2017/ajaxcalls/PosterInfo.asp?efp=S1lVTUxLQVozODMy&PosterID=115995&rnd=0.8782085).

Risk factors for gastric cancer in the excluded stomach are not well established. It is unclear if the loss of excess weight, maintained over a long period, or the lack of food contact with excluded stomach could reduce the higher risk [1].  Duodenal reflux is present in up to 36% of patients subjected to gastric bypass [9]. The effects of biliary salts on gastric mucosa in the development of gastritis, intestinal metaplasia, and cancer in patients undergoing subtotal gastrectomy have been reported [10].

It is important to consider family history, the presence of certain familiar disorders, such as hereditary non-polyposis colon cancer and Li-Fraumeni syndrome, and precancerous lesions, such as adenomatous polyps, dysplasia, intestinal metaplasia, and Ménétrier’s disease, while evaluating patients for gastric bypass. In these high-risk patients, resection of the bypassed stomach can be considered at the time of RYGB surgery as has been reported with intestinal metaplasia [11]. However, the risks associated with a routine gastric resection in patients undergoing gastric bypass outweighs the benefits.

Preoperative esophagogastroduodenoscopy (EGD) is therefore mandatory before bariatric surgery. However, clear recommendations on postoperative surveillance EGD has not been established [6].

Symptoms of gastric cancer in the excluded stomach are usually non-specific and may include anorexia, epigastric pain, nausea, vomiting, and anemia, as well as further weight loss following a period of stability or weight gain.

While altered anatomy after RYBG surgery may pose technical difficulties in visualizing the excluded stomach by conventional endoscopy alone, a variety of minimally invasive techniques can be used to overcome this difficulty, such as ADBE, percutaneous endoscopy, virtual gastroduodenoscopy, laparoscopic transgastric endoscopy, and EUS [12-16].

In our cases, we used ADBE and EUS-guided visualization with biopsies to access the excluded stomach. ADBE is a well-recognized technique used to investigate the gastrointestinal tract up to many feet. It can be used effectively to access the bypassed stomach in patients with gastric bypass surgery but is time-consuming [12]. EUS is a safe and quick procedure in general and can be used as an initial modality in patients with suspected malignancy of the excluded stomach or other surgically altered anatomy [16].

## Conclusions

Patients with prior RYGB presenting with new-onset unexplained symptoms should prompt evaluation, including an adequate visualization of the excluded stomach, using cross-sectional imaging and complemented with advanced endoscopy techniques.

## References

[1] Escalona A, Guzmán S, Ibáñez L (2005). Gastric cancer after Roux-en-Y gastric bypass. Obes Surg.

[2] Steinbrook R (2004). Surgery for severe obesity. N Engl J Med.

[3] Torre LA, Bray F, Siegel RL (2015). Global cancer statistics, 2012. CA Cancer J Clin.

[4] Inoue H, Rubino F, Shimada Y (2007). Risk of gastric cancer after Roux-en-Y gastric bypass. Arch Surg.

[5] Lord R, Edwards P, Coleman M (1997). Gastric cancer in the bypassed segment after operation for morbid obesity. ANZ J Surg.

[6] Orlando G, Pilone V, Vitiello A (2014). Gastric cancer following bariatric surgery: a review. Surg Laparosc Endosc Percutan Tech.

[7] de Martel C, Forman D, Plummer M (2013). Gastric cancer: epidemiology and risk factors. Gastroenterol Clin North Am.

[8] Renehan AG, Tyson M, Egger M (2008). Body-mass index and incidence of cancer: a systematic review and meta-analysis of prospective observational studies. Lancet.

[9] Sundbom M, Hedenström H, Gustavsson S (2002). Duodenogastric bile reflux after gastric bypass: a cholescintigraphic study. Dig Dis Sci.

[10] Sugiyama Y, Sohma H, Ozawa M (1987). Regurgitant bile acids and mucosal injury of the gastric remnant after partial gastrectomy. Am J Surg.

[11] Voellinger DC, Inabnet WB (2002). Laparoscopic Roux-en-Y gastric bypass with remnant gastrectomy for focal intestinal metaplasia of the gastric antrum. Obes Surg.

[12] Kuga R, Safatle-Ribeiro AV, Faintuch J (2007). Endoscopic findings in the excluded stomach after Roux-en-Y gastric bypass surgery. Arch Surg.

[13] Gill KR, McKinney JM, Stark ME, Bouras EP (2008). Investigation of the excluded stomach after Roux-en-Y gastric bypass: the role of percutaneous endoscopy. World J Gastroenterol.

[14] Silecchia G, Catalano C, Gentileschi P (2002). Virtual gastroduodenoscopy: a new look at the bypassed stomach and duodenum after laparoscopic Roux-en-Y gastric bypass for morbid obesity. Obes Surg.

[15] Richardson JF, Lee JG, Smith BR (2012). Laparoscopic transgastric endoscopy after Roux-en-Y gastric bypass: case series and review of the literature. Am Surg.

[16] Wilson JA, Hoffman B, Hawes RH, Romagnuolo J (2010). EUS in patients with surgically altered upper GI anatomy. Gastrointest Endosc.

